# The incidence of breast cancer in the General Practice Research Database compared with national cancer registration data

**DOI:** 10.1054/bjoc.2000.1493

**Published:** 2000-11-22

**Authors:** J A Kaye, L E Derby, M del Mar Melero-Montes, M Quinn, H Jick

**Affiliations:** Harvard School of Public Health, 677 Huntington Avenue, Boston, MA, 02115, USA; Boston Collaborative Drug Surveillance Program, Boston University School of Medicine, 11 Muzzey Street, Lexington, MA, 02421, USA; National Cancer Intelligence Centre, Office for National Statistics, 1 Drummond Gate, London, SW1V 2QQ, UK

**Keywords:** breast cancer, incidence rates, General Practice Research Database, cancer registries, mammography, screening

## Abstract

Breast cancer incidence rates in the UK from 1990 to 1996 among women aged 35–69 estimated from the General Practice Research Database (GPRD) were closely similar to those reported by the Office for National Statistics from cancer registration data (ONS). The GPRD is a valuable and up-to-date resource for further study of the incidence of breast cancer in the UK as well as changes in cancer treatment and their relation to survival trends. © 2000 Cancer Research Campaign http://www.bjcancer.com


*British Journal of Cancer* (2000) **83**(11), 1556–1558
© 2000 Cancer Research Campaign
doi: 10.1054/ bjoc.2000.1493, available online at http://www.idealibrary.com on IDEAL®
http://www.bjcancer.com

Short Communication

### The incidence of breast cancer in the General Practice Research Database compared with national cancer registration data

**JA Kaye,[1,2] LE Derby,[2] M del Mar Melero-Montes,[2] M Quinn[3] and H Jick[2]**

[1]Harvard School of Public Health, 677 Huntington Avenue, Boston, MA 02115, USA; [2]Boston Collaborative Drug Surveillance Program, Boston University School of Medicine, 11 Muzzey Street, Lexington, MA 02421, USA; [3]Director, National Cancer Intelligence Centre, Office for National Statistics, 1 Drummond Gate, London, SW1V 2QQ, UK

Summary Breast cancer incidence rates in the UK from 1990 to 1996 among women aged 35–69 estimated from the General Practice Research Database (GPRD) were closely similar to those reported by the Office for National Statistics from cancer registration data (ONS). The GPRD is a valuable and up-to-date resource for further study of the incidence of breast cancer in the UK as well as changes in cancer treatment and their relation to survival trends. © 2000 Cancer Research Campaign http://www.bjcancer.com

**Keywords:** breast cancer; incidence rates; General Practice Research Database; cancer registries; mammography; screening

### INTRODUCTION

In the UK, incidence rates of breast cancer reported by the National Cancer Intelligence Centre at the Office for National Statistics (ONS; formerly the Office of Population Censuses and Surveys) among women aged 50–64 increased after 1988 (Quinn and Allen, 1995) when widespread mammographic screening of women in this age range was introduced (Department of Health and Social Security, 1986).

The General Practice Research Database (GPRD) has been used extensively in pharmacoepidemiologic studies of cancer and other illnesses (Walley and Mantgani, 1997; Jick, 1997). We estimated the age-adjusted annual incidence rates of breast cancer in women aged 35–69 during the years 1990–1996 in the GPRD and compared these rates to those from the ONS (Quinn et al, 1999).

### MATHERIALS AND METHODS

Detailed descriptions of the cancer registration system in the UK have been published elsewhere (ONS, 1999; Quinn and Allen, 1995).

The General Practice Research Database (GPRD) contains records for some 3 million people in the UK. All clinical data from the GPRD are provided to the researchers with patients identified only by unique numbers. We selected for study all 263 practices that had been continuously active since their date of entry into the GPRD and had updated their data to the end of September 1997. None of the practices contributing data to this study joined the GPRD after 1993.

*Received 12 June 2000*
*Revised 21 August 2000*
*Accepted 21 August 2000*

*Correspondence to:* JA Kaye

Cases were identified by three selection processes and then confirmed (or otherwise) by review of computer records. The first was to select records of women who had a first-time diagnostic code for breast carcinoma. The second was to select records of women who were prescribed tamoxifen for the first time in a given year but did not have a specific diagnosis of breast carcinoma. Finally, we selected records of women with a set of characteristics (mastectomy or breast lump or biopsy followed within 6 months by a recorded diagnosis of carcinoma (type unspecified) or radiation therapy) which indicated that she may have had breast cancer even if the diagnosis was not recorded and tamoxifen was not prescribed.

We then reviewed the computer records of all women selected by the above process to determine whether each was an incident case of breast carcinoma. For a woman to be confirmed as an incident case, we required that she have a breast lump or mammographic abnormality recorded immediately before the diagnosis of breast cancer, and a subsequent mastectomy, lumpectomy, or fine needle aspiration with comments indicating a finding of cancer. We also included cases in the absence of the above criteria if the diagnosis of breast cancer was followed by the initiation of chemotherapy or radiation therapy or if there were other comments recorded that justified identifying the case as incident. We excluded women with a history of breast cancer before the study period. Only confirmed cases were analysed further. Although there is no distinct diagnostic code for breast carcinoma in situ, this histology was noted in free text comments in approximately 5% of records of women aged 50–64.

We classified confirmed incident breast cancer cases by year of diagnosis, by age at diagnosis and by geographic region. Crude and age-specific annual incidence rates were calculated using the total number of women registered in the GPRD during each year of the study. Directly age standardized incidence rates were calculated using the European standard population (dos Santos Silva, 1999). Statistical calculations were performed using Stata version 6.0 (Stata Corp., College Station, TX).

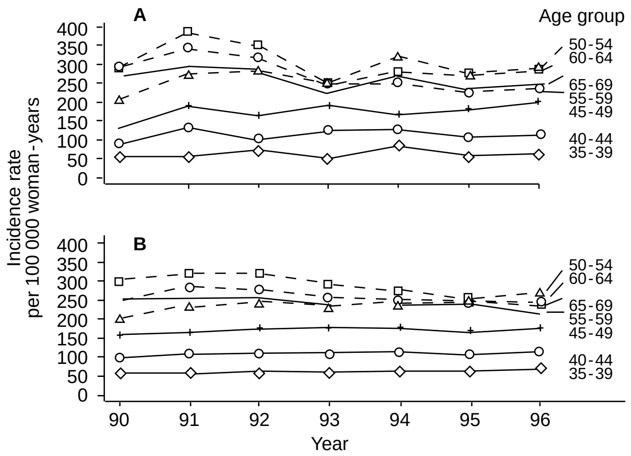

**Figure 1** Age-specific incidence rates by year of diagnosis in the GPRD (**A**) and ONS data (**B**). The NHS Breast Cancer Screening Programme targeted women in the age groups indicated by dashed lines (50–54, 55–59, and 60–64).

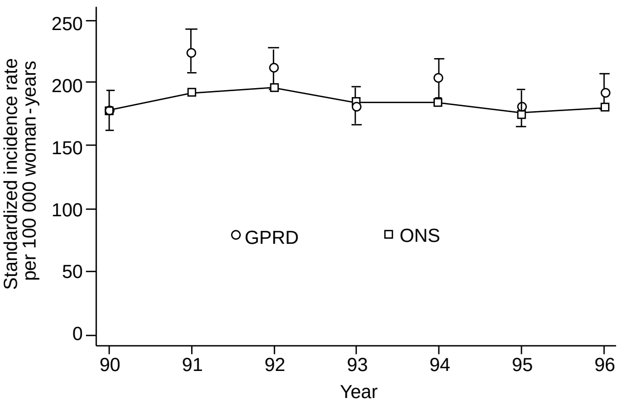

**Figure 2** GPRD and ONS breast cancer incidence rates among women aged 35–69 directly standardized to the European standard population. Error bars indicate 95% confidence intervals for the GPRD rates.

### RESULTS

From 1990 to 1996, 417 891 women aged 35 to 69 contributed a total of 2 181 296 woman-years. We found 4248 incident cases of breast cancer. 3935 (93%) women had a coded diagnosis of breast carcinoma recorded in the GPRD for the first time during one of these years. An additional 266 (6%) women without a diagnosis of breast carcinoma were prescribed tamoxifen for the first time during the study and were found to be incident cases of breast cancer upon review of their computer records. Among women who neither had a diagnosis of breast carcinoma nor received tamoxifen, 47 (1%) had a mastectomy or either a breast lump or biopsy followed within 6 months by either a diagnosis of carcinoma (unspecified type) or radiation therapy and were considered to be incident cases of breast cancer.

GPRD breast cancer incidence rates increased with increasing age among women aged 35–39, 40–44, 45–49 and 50–54 (Figure 1A). ONS data show the same patterns (Figure 1B). However, the incidence rates for women aged 55–59 in the GPRD were higher than the rates for women aged 65–69 from 1990 to 1993, and the incidence rates for women aged 60–64 were higher than the rates for women aged 65–69 in every year of the study. The ONS data are similar in that incidence rates among the age groups targeted for screening (50–64) were similar to or higher than those for women aged 65–69 during most years of the study (Figure 1B). There was a sustained increase in incidence among women aged 50–54 in both the GPRD and ONS data (Figures 1A, 1B).

The directly age standardized breast cancer incidence rates from the GPRD and the ONS data are shown in Figure 2; the rates are similar throughout the period, except that the GPRD rates for 1991 and 1994 are significantly higher than the ONS rates. Both the GPRD and ONS data suggest a decrease in incidence rates in 1993 but the changes are not statistically significant.

The annual incidence of breast cancer was examined for 11 areas of the UK (the 8 health regions of England, Wales, Scotland and Northern Ireland). In a Poisson regression model adjusting for age, the incidence of breast cancer was not significantly different among the 11 regions (results not shown).

### DISCUSSION

Our study found that breast cancer incidence rates among women aged 35–69 in the GPRD were closely similar to those reported by the ONS. The slightly higher incidence rates in the GPRD in some years of the study are probably due to the inclusion in the GPRD of in situ carcinomas, while the ONS data include only invasive cancers. The higher incidence rates for women aged 55–59 and 60–64 compared with women aged 65–69 are likely to be due to the NHS Breast Screening Programme.

Although the initial round of mammographic screening was planned to take place in the UK from 1988 to 1991, the acceptance rate for screening across the UK averaged 71%, and only 72% of screening programmes reached the target 70% acceptance rate (Chamberlain et al, 1993). Because some slippage in the screening programme schedule also occurred (Faux et al, 1998), incidence rates in 1991 among women invited for screening were still increasing. Increases in incidence among women aged 50–54 relative to those aged 55–59 may have resulted from depletion of screen-detectable tumours among the older screened women and continued entry into the study population of newly screened women aged 50–54.

Only a few small studies have provided validation of results from the UK cancer registries, and only a small number of breast cancers have been included in these studies (Huggett, 1995; Brewster et al, 1997). In one study in the mid-1980s, only 36 of 50 (72%) cases of breast cancer in a cohort of 4544 taking hormone replacement therapy had been included in the national registry system (Hunt and Coleman 1987). In another report, 138 of 150 (92%) breast cancers reported to investigators of the Oxford-FPA contraceptive study were registered (Villard-Mackintosh et al, 1988). In a more recent study, 535 of 599 (89%) breast cancers diagnosed at one hospital in the Trent region were registered within one year (Stotter et al, 2000).

Although protection of personal identity in the GPRD precludes identification of the subjects diagnosed with breast cancer and direct comparison with cancer registry data, the close similarity of incidence rates between the GPRD data and recent ONS data (Quinn et al, 1999), the generally low proportion of cases recorded

*© 2000 Cancer Research Campaign*

solely from the information on a death certificate (Parkin et al, 1994), and the coherence of the national data on incidence, mortality, and survival all provide evidence that the GPRD and the national registration data are similarly, and substantially, complete. The GPRD data are updated annually and can be used to confirm national trends in cancer incidence at relatively low cost because they are already being collected to document ongoing medical care.

While continuation of dedicated cancer registration systems remains vital to cancer control and public health, large-scale medical databases such as the GPRD can also provide timely and accurate information on cancer incidence. The richness of medical data in the GPRD also presents the possibility of further study of cancer etiology, morbidity, and survival.

### ACKNOWLEDGEMENTS

We wish to thank the participating general practitioners for their excellent cooperation and generous help, and Alexander M Walker of the Harvard School of Public Health and Susan S Jick of the Boston Collaborative Drug Surveillance Program for their thoughtful review of the manuscript. The Boston Collaborative Drug Surveillance Program is supported in part by grants from AstraZeneca, Berlex Laboratories, Boots Healthcare International, Bristol-Myers Squibb Pharmaceutical Research Institute, Glaxo Wellcome Inc, Hoffmann-La Roche Ltd, Janssen Pharmaceutica Products, LP, RW Johnson Pharmaceutical Research Institute, McNeil Consumer Products Company and Novartis Farmacéutica SA.

### REFERENCES

Brewster DH, Crichton J, Harvey JC and Dawson G (1997) Completeness of case ascertainment in a Scottish regional cancer registry for the year 1992. *Public Health* **111**: 339–343

Chamberlain J, Moss SM, Kirkpatrick AE, Michell M and Johns L (1993) National Health Service breast cancer screening programme results for 1991–2. *BMJ* **307**: 353–356

Department of Health and Social Security (1986) Breast cancer screening: report to the health ministers of England, Wales, Scotland and Northern Ireland. London: HMSO, 1986 (Forrest report.)

dos Santos Silva I (1999) *Cancer Epidemiology: Principles and Methods*. Lyons: International Agency for Research on Cancer

Faux AM, Lawrence GM, Wheaton ME, Wallis MG, Jeffery CL and Griffiths RK (1998) Slippage in the NHS breast screening programme: an assessment of whether a three year screening round is being achieved. *J Med Screen* **5** 88–91

Huggett C (1995) Review of the quality and comparability of data held by regional cancer registries. Bristol: Bristol Cancer Epidemiology Unit

Hunt K and Coleman MP (1987) The completeness of cancer registration in follow-up studies – a cautionary note. *Br J Cancer* **56**: 357–359

Jick H (1997) A database worth saving. *Lancet* **350**: 1045

Office for National Statistics (1999) *Cancer statistics – registrations, England & Wales, 1993* ONS Series MB1 no. 26, London: The Stationery Office

Parkin DM, Chen VW, Ferlay J, Galceran J, Storm HH and Whelan SL (1994) *Comparability and Quality Control in Cancer Registration*. IARC Technical Report No. 19. Lyons, International Agency for Research on Cancer

Quinn M and Allen E (1995) on behalf of the United Kingdom Association of Cancer Registries. Changes in incidence of and mortality from breast cancer in England and Wales since introduction of screening. *BMJ* **311**: 1391–1395

Quinn MJ, Babb PJ, Jones J and Brock A (1999) *Report: registrations of cancer diagnosed in 1993–1996 England and Wales*. Health Statistics Quarterly No. 4. London: Office for National Statistics

Stotter A, Bright N, Silcocks PB and Botha JL (2000) Effect of improved data collection on breast cancer incidence and survival: reconciliation of a registry with a clinical database. *BMJ* **321**: 214

Villard-Mackintosh L, Coleman MP and Vessey MP (1988) The completeness of cancer registration in England: an assessment from the Oxford-FPA contraception study. *Br J Cancer* **58**: 507–511

Walley T and Mantgani A (1997) The UK General Practice Research Database. *Lancet* **350**: 1097–1099

 *© 2000 Cancer Research Campaign*

